# Crossing Boundaries: Global Reorientation Following Transfer From the Inside to the Outside of an Arena

**DOI:** 10.1037/xan0000206

**Published:** 2019-05-09

**Authors:** Matthew G. Buckley, Luke J. Holden, Stuart G. Spicer, Alastair D. Smith, Mark Haselgrove

**Affiliations:** 1 School of Applied Social Sciences, De Montfort University; 2 School of Psychology, University of Nottingham; 3 School of Psychology, University of Plymouth; 4 School of Psychology, University of Nottingham

**Keywords:** spatial learning, geometric module, associative learning, navigation, environmental boundary

## Abstract

In 2 spatial navigation experiments, human participants were asked to find a hidden goal (a WiFi signal) that was located in 1 of the right-angled corners of a kite-shaped (Experiment 1) or a cross-shaped (Experiment 2) virtual environment. Goal location was defined solely with respect to the geometry of the environment. Following this training, in a test conducted in extinction, participants were placed onto the outside of the same environments and asked to locate the WiFi signal. The results of both experiments revealed that participants spent more time searching in regions on the outside of the environments that were closest to where the WiFi signal was located during training. These results are difficult to explain in terms of analyses of spatial navigation and reorientation that emphasize the role of local representational encoding or view matching. Instead, we suggest that these results are better understood in terms of a global representation of the shape of the environment.

In order to navigate efficiently, organisms must maintain a sense of direction within the environment. If an organism becomes disoriented, for some reason, a process of reorientation must occur in order for the direction of travel to be reestablished. In a seminal article published over three decades ago, Cheng (1986) observed that rats reoriented using the shape provided by the boundary walls of an environment. In his experiment, Cheng (1986) trained rats to find buried food in the corner of a rectangle-shaped arena, which also contained a unique landmark in each corner. Following this training, the landmarks were removed from the rewarded corner, and the corner diagonally opposite, and it was observed that rats searched for food in the correct corner, and the geometrically equivalent corner that was diagonally opposite. Interestingly, the presence of unambiguous landmark cues did not preclude learning about the ambiguous geometry of the environment. On the basis of these findings, Cheng (1986) proposed that organisms encode a representation of the global-shape of an environment in a dedicated geometric module that is immune to the influence of nonshape cues.

The hypothesis that learning about the shape of an environment is encapsulated, or modular, has received much empirical attention (see Cheng, 2008; Twyman & Newcombe, 2010 for reviews). In comparison, the nature of the representation that may support shape-based reorientation has been much less researched, despite the fact that a variety of organisms can use the shape of an environment to reorient, including, fish (Sovrano, Bisazza, & Vallortigara, 2002), chicks (Vallortigara, Zanforlin, & Pasti, 1990), mountain chickadees (Gray, Bloomfield, Ferrey, Spetch, & Sturdy, 2005), pigeons (Kelly, Spetch, & Heth, 1998), rats (Hayward, Good, & Pearce, 2004), rhesus monkeys (Gouteux, Thinus-Blanc, & Vauclair, 2001), as well as children (e.g., Hermer & Spelke, 1994, 1996) and adult humans (Redhead & Hamilton, 2007, 2009). According to global theories of shape-based reorientation, a representation of the entire shape of an environment is encoded, and it is this representation that guides reorientation behavior. As noted previously, Cheng (1986) proposed that shape information is processed in a dedicated module that supports encoding of only a global representation (see also: Wang & Spelke, 2002, 2003), and this position was championed by Gallistel (1990) who claimed that, when disoriented, animals reorient on the basis of the global shape of the environment. A similar conclusion was also reached by Cheng and Spetch (1998) who, when discussing the findings reported by Cheng (1986), claimed that the animals used only the broad shape of the environment to find the buried food (p. 15; see also Cheng, Huttenlocher, & Newcombe, 2013; and Cheng & Newcombe, 2005).

The notion that animals encode global-shape representations has not gone unchallenged, however. According to local theories of shape-based reorientation, animals may encode, for example, the relative wall lengths that are provided by the conjunction of two walls (Pearce, Good, Jones, & McGregor, 2004: see also McGregor, Jones, Good, & Pearce, 2006; Pearce, 2009). This being the case, it is possible to explain the findings reported by Cheng (1986) by assuming that rats learn the location of the buried food on the basis of the local-shape information that is present only at the rewarded corner, and not on the basis of the global shape of the environment. According to this analysis, rats associate a goal with relative wall length information, such as the view of a short wall is to the left of long wall (Pearce et al., 2004). Crucially, in a rectangle, the baited corner, and the corner diagonally opposite are identical in local shape properties. Rats navigating on the basis of local-shape information, then, would be expected to visit the diagonally opposite corner, as was observed in the experiments conducted by Cheng (1986: see also: Margules & Gallistel, 1988).

Evidence in favor of local-shape encoding has come from shape transformation experiments in which, following training, the global-shape of an environment is changed while some of the local-shape information is preserved. For instance, Lew et al. (2014) trained adult humans to find a hidden goal in a right-angled corner of a kite-shaped virtual environment, before transferring them to a rectangle-shaped virtual environment. While the global shapes of these two environments differed, both the kite- and rectangle-shaped environments contained at least one right angled corner where a short wall was to the left of a long wall, and at least one right angled corner where a short wall was to the right of a long wall. Following training in the kite-shaped environment, and upon being placed into the rectangle-shaped environment, Lew et al. (2014) observed that participants preferentially searched in the corner of the arena that shared the same local-shape properties that signaled the goal location in the kite-shaped environment. Given that the global-shape of the two environments in the experiment were different, this preference could only have been driven by local-shape information. (see also Esber, McGregor, Good, Hayward, & Pearce, 2005; Pearce et al., 2004; Poulter, Kosaki, Easton, & McGregor, 2013).

It is important to note, however, that evidence that organisms encode local-shape information does not constitute evidence against the encoding of global-shape information. For instance, in the training stage of the experiment conducted by Lew et al. (2014), it is possible that participants encoded *both* the local- and the global-shape properties of the kite-shaped environment. At test, however, the global representation of the kite-shaped training environment would be incongruent to the, now, rectangle-shaped test arena. Consequently, any global representation encoded by participants during training would be of little worth in guiding navigation during test; thus, forcing them to reorient on the basis of the local-shape properties that were preserved between the training and testing environments. That being said, evidence that organisms encode the global shape of an environment, and use this representation to guide reorientation behavior, has not been particularly forthcoming. Part of the problem in providing evidence for global-shape encoding is that it is not entirely clear how to dissociate reorientation based on global-shape information from local-shape information. As the shape transformation experiments discussed above demonstrate, it is possible to change the global-shape of an environment and, at the same time, preserve local-shape cues; however, it is more difficult to conceive of a transformation in which the local-shape cues are changed, while at the same time the global shape of the environment is preserved.

One possible strategy to dissociate behavior based on global-shape information from local-shape information, however, is to employ a perspective transformation, in which participants are transferred from the inside to the outside of an arena. Consider training in which a participant is trained in a kite-shaped arena to locate a hidden goal at the inside corner where a short wall is to the left of a long wall. When placed on the outside of the same arena, the view of the goal corner is a short wall to the right of a long wall; thus, the relative lengths of the left- and right-sided walls are reversed from training (see Figure 1, Panel A). Consequently, reorienting on the basis of viewpoint dependent local-shape representations (Pearce et al., 2004; Stürzl, Cheung, Cheng, & Zeil, 2008; see also Cheng et al., 2013; Nardini, Thomas, Knowland, Braddick, & Atkinson, 2009) that are defined only in terms of relative wall lengths (e.g., a short left wall and a long right wall) would not lead the participant to the outside of the corner that contained the hidden goal during training. In contrast, as global-shape representations are thought to be viewpoint independent (Burgess, 2006; Cheng, 1986), and because the overall shape of an environment does not change depending on whether the walls are viewed from the inside or the outside, reorientation on the basis of a representation of the global-shape would lead the participant to the outside of the corner paired with the goal during training.[Fig-anchor fig1]

In a recent series of experiments that employed perspective transformations, Buckley, Smith, and Haselgrove (2016b, 2019) provided evidence that humans can use global-shape information during reorientation. In Buckley et al.’s (2016b) Experiment 1, participants were trained to find a hidden goal (a WiFi signal) that was located at a right-angled corner inside a virtual kite-shaped arena. Following this training, participants were given a single test trial on the outside of the kite-shaped environment, and were again required to search for the WiFi signal which, unbeknownst to the participant, was not present. During this test, participants preferentially searched on the outside of the corner that contained the WiFi signal; a behavior that is not consistent with reorientation based on local-shape information, but that is in keeping with the idea that reorientation *can* be based upon a global representation of the shape of the arena (see also Lourenco & Huttenlocher, 2007; Lourenco, Huttenlocher, & Vasilyeva, 2005).

The purpose of the experiments reported here was to further explore whether reorientation behavior can be controlled by local-shape information following a perspective transformation, or whether this behavior continues to be controlled by a global-shape representation following transfer from one side of a boundary to the other. In particular, we investigated the possibility that local-shape information fails to control reorientation following a perspective-transformation because of a decrement in generalization. In Experiment 1 reported by Buckley et al. (2016b), participants were trained inside a virtual building with wooden floors, and cream colored walls, but at test participants searched on the outside of the building with a grassy texture applied to the floor, and a brick texture on the walls. The change in appearance of the environment may be an important determinant of spatial behavior, as it has previously been demonstrated that removing some of the landmarks from an array of proximal landmarks disrupts the search behavior of human participants trained to find a hidden goal in a virtual watermaze (Sansa, Aznar-Casanova, Rodríguez, & Chamizo, 2019). If local-shape encoding is particularly prone to generalization decrement, then changes in the appearance of the environment may cause the representation to lose some control over reorientation behavior. Consequently, in the test phase of the experiments reported by Buckley et al. (2016b), participants may have relied less on the local-shape cues for reorientation, instead favoring to reorient on the basis of global-shape cues. In the present Experiment 1, therefore, we replicated Experiment 1 reported by Buckley et al. (2016b), but with the addition of a group for whom the inside and the outside of the arena were better matched in terms of visual appearance. To foreshadow our results, we again observed reorientation behavior that was consistent with a global, rather than a local, representation of environmental shape.

In Experiment 2, we adopted a more complete definition of local-shape information in order to further reduce the generalization decrement suffered by local-shape cues between training and test. In order to reorient on the basis of this local-shape information following a shift from, say, the inside of a kite-shaped boundary to the outside of the same boundary (Buckley et al., 2016b, Experiment 1a, and Experiment 1 of the present article), participants are required to transfer the relative wall length information from a 90° corner to 270° corner. There is, however, evidence that organisms encode the angular information that is provided by the conjunction of two walls (Lubyk, Dupuis, Gutiérrez, & Spetch, 2012; Sturz, Forloines, & Bodily, 2012; Tommasi & Polli, 2004), and so it may be unreasonable to expect that relative wall length information will transfer across such different angles. Moreover, given that animals have been shown to encode angular information, it is likely that local-shape representations include not only relative wall lengths, but also the angle that is created at the join of the two walls. Consequently, in order to provide a fair test of reorientation based on local-shape cues following a perspective transformation, it is necessary to study reorientation using a boundary-shape that preserves the entire local-shape cue between training and test. We achieved this in Experiment 2 by examining reorientation using a novel cross-maze. Here, the exact local-shape cue that signaled the goal location during training on the inside of the environment was also present on the outside of the environment but, crucially, this location was spatially dissociated from reorientation behavior that would be based on a global-shape representation.

## Experiment 1

**Figure 1 fig1:**
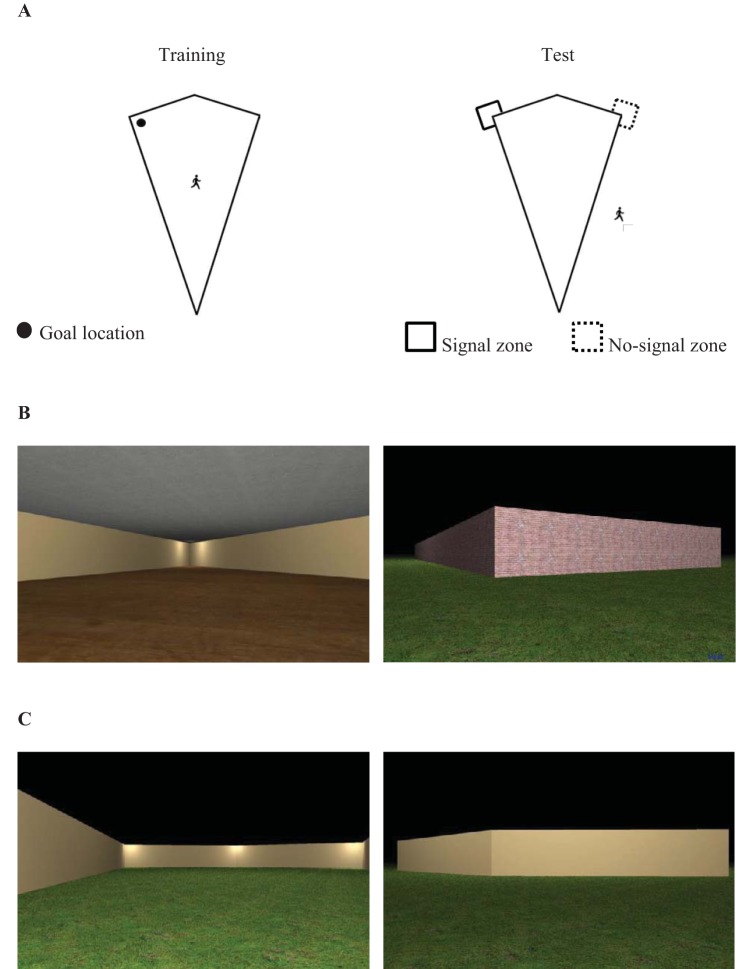
Panel A displays schematic views of the training and test environments for the same and different groups of Experiment 1. The black circle represents a goal location, and square search zones are superimposed on the diagram of the test environment. The location of the person indicates whether participants were navigating on the inside, or the outside, of the arena. Panel B displays examples of the training (left) and test (right) environments for the different group. Panel C displays the training (left) and test (right) environments for the same group.

The purpose of Experiment 1 was to investigate the impact of matching the appearance of the surface textures of the inside of the environment that defined the location of the hidden goal during training to the appearance of the surface textures of the outside of same-shaped environment at test. Participants in the present experiment were trained to find a hidden WiFi signal in, for example, the right-angled corner of a kite-shaped arena where the left wall was longer than the right wall. Following training, participants were placed on the outside of the kite-shaped environment, in the absence of any hidden goal, and allowed to search for 120 s. Here, we measured time spent in a signal zone that was located around the outside of the right-angled corner that was rewarded in training, and also time spent in a no-signal zone that surrounded the other right-angled corner of the kite-shaped arena (see Figure 1, Panel A). Participants in the *different* group were trained with the same arena as employed by Buckley et al. (2016b); thus, a wooden texture was applied to the floor, the walls were cream in color, and the ceiling was a uniform gray during the training stage of the experiment. During the test phase, a grass texture was applied to the floor, a brick texture was applied to the walls, and the sky was a uniform black expanse (see Figure 1, Panel B). For participants in the *same* group, however, both the training and testing environments had a grass texture applied to the floor, cream colored walls, and a black sky (See Figure 1, Panel C). Our first expectation was to reproduce the effects observed by Buckley et al. (2016b) in the different group; thus, following training inside the kite-shaped arena, participants should preferentially explore the signal zone corner over the no-signal zone corner at test. The question of interest was whether participants in the same group would show any evidence of reorientation based on local-shape information when the visual information was better matched between training and testing, and thus the generalization decrement suffered by local-shape cues following the transfer across the boundary was reduced. This would be indexed by participants spending less time in the signal zone relative to the different group, and instead more time in the no-signal zone that matched the local-shape cue, at least in terms of the relative wall-length information (Pearce et al., 2004), that was rewarded during training.

### Method

#### Participants

32 students were recruited from the University of Nottingham (23 female), aged between 21 and 45 years (*M* = 23.81, *SD* = 4.43), and were given £5 in return for participation. Participants were randomly allocated to an experimental group, with the stipulation that there were 16 participants in each group. All participants provided fully informed consent before commencing the experimental procedure, and the study was ratified by the School of Psychology Research Ethics Committee (University of Nottingham).

#### Materials

MazeSuite software (Ayaz, Allen, Platek, & Onaral, 2008; www.mazesuite.com) was used to construct and display the virtual environments, which participants viewed from a first-person perspective. The virtual environments were displayed on an Apple Macintosh model A1224 (EMC2133) with a screen of 274 mm × 434 mm. Assuming a walking speed similar to that in the real world (2 m/s), the perimeter of both of the kite-shaped arenas was 72 m, with the small walls being 9 m, and the long walls 27 m, in length. The height of the walls in both arenas was approximately 2.5 m. The arenas contained two right-angled corners, with the remaining two angles being 143.14° and 36.86°. Within the environments, the hidden goals were square-shaped regions (1.08 m × 1.08 m) that were always placed 2.48 m away from the walls of the arena, along on a notional line that bisected the corner.

For participants in the same group, the kite-shaped training and test environments were both built from cream-colored walls that, using the 0–255 RGB scale employed by MazeSuite, were defined as 204, 178, 127. In both training and testing environments, a grass texture was applied to a 780 m × 780 m floor, and the sky was rendered as a uniform black expanse. For participants in the different group, the training environment had a wooden texture was applied to the floor, cream walls, and a uniform gray texture was applied to the ceiling (see Figure 1). In contrast, the testing environment had a grass texture applied to the floor, brick walls, and a black sky.

#### Procedure

After signing a standard consent form, participants in both groups were given the following standard set of instructions:

This study is assessing human navigation using a computer generated virtual environment. During this experiment, you will complete 16 trials. In each trial, you will be placed into a room that contains a WiFi hot spot. Your aim is to end the trials as quickly as possible by walking into the hot spot.

You will view the environment from a first person perspective, and be able to walk into the hot spot from any direction using the cursor keys on the keyboard. Once you’ve found the hot spot a congratulatory message will be displayed and you should hit enter when you’re ready to begin the next trial. You will always be in the center of the arena when a trial begins, but the direction in which you face at the start of each trial will change.

To start with, you may find the hot spot is difficult to find. The hot spot does not move though, so it is possible to learn its specific location as the experiment goes along. It’s a good idea to fully explore the environment on the first few trials to become aware of your surroundings. This should help you in learning where the hidden hot spot is.

This session should take around 20 min. If at any point you wish to stop this session, please notify the experimenter and you’ll be free to leave without having to give a reason why. Your results will be saved under an anonymous code, and kept confidential throughout.

During the experiment, participants sat not more than 50 cm from the screen, and navigated through the virtual environments using the cursor keys. Presses on the “up” and “down” cursor keys permitted the participant to move forward and backward within the arena, respectively, while presses on the “left” and “right” cursor keys permitted the participant to rotate counterclockwise and clockwise within the environment, respectively.

During the 16 acquisition trials, participants began each trial at a point located halfway between the apex and obtuse corners, and the direction in which participants began facing was randomized (between 0° and 359°) for every trial. Once participants had navigated to the hidden goal they could no longer move, and a congratulatory message (*WiFi Connected!*) was displayed on screen using the default font and character size in MazeSuite. Participants pressed enter to begin the next trial. There was no time limit for any acquisition trials, thus, each trial ended only when the hidden goal was found. If, however, 120 s elapsed on a given trial a white flag appeared at the goal location. A counter was presented in the lower-right corner of the screen that indicated to participants the time elapsed (in seconds) within each trial. For both groups in the experiment, the location of the hidden goal was counterbalanced such that eight participants within each group were required to navigate to a right-angled corner where a long wall was to the left of a short wall, while the remaining eight participants in each group were required to navigate to a right-angled corner where a long wall was to the right of a short wall.

Having completed 16 acquisition trials, participants received the following instructions prior to the test trial:

In the next trial, you will again have to locate a WiFi signal. The location of the WiFi signal hasn’t changed, so it will be in the same location as before.

However, you will be navigating around the outside of the building. As the WiFi signal will be traveling through the walls of the building, it will be a bit weaker, and so it may be harder to locate.

Press enter to start.

For participants in both groups, pressing enter began a 120-s test trial in which participants were placed on the outside of an arena that contained no hidden goals. Participants began the test trial facing one of the four walls of the kite-shaped arena, and were located 3.15 m from the center of the wall, along a notional line running perpendicular to the wall. There were four possible start locations for the test trial, and each location was used twice in every set of eight participants previously described. To measure behavior during test trials, we recorded the time spent within L-shaped search zones (long sides 6.48 m, short sides 3.24 m) that wrapped around the right-angled corners of the environment (see Figure 1, Panel A). The signal zone was located at the right angled corners of an environment that had previously contained the hidden goal, and the no-signal zone were located at the right angled corners of an environment that did not previously contain the hidden goal. Assessing spatial behavior during extinction tests in such a manner is common in both animal (e.g., McGregor, Horne, Esber, & Pearce, 2009), and human (e.g., Redhead & Hamilton, 2009) experiments.

### Results

In both experiments reported here, we treat data with an analysis of variance (ANOVA), and report partial eta squared (η_p_^2^) to estimate effect sizes. In order to generate confidence intervals that are congruent with the outcomes of an ANOVA that adopts .05 as the criterion for significance, we calculated 90% confidence intervals around η_p_^2^ (Steiger, 2004). Here, the confidence interval surrounding an effect size will only exclude zero when the corresponding *p* value < .05.

#### Training

**Figure 2 fig2:**
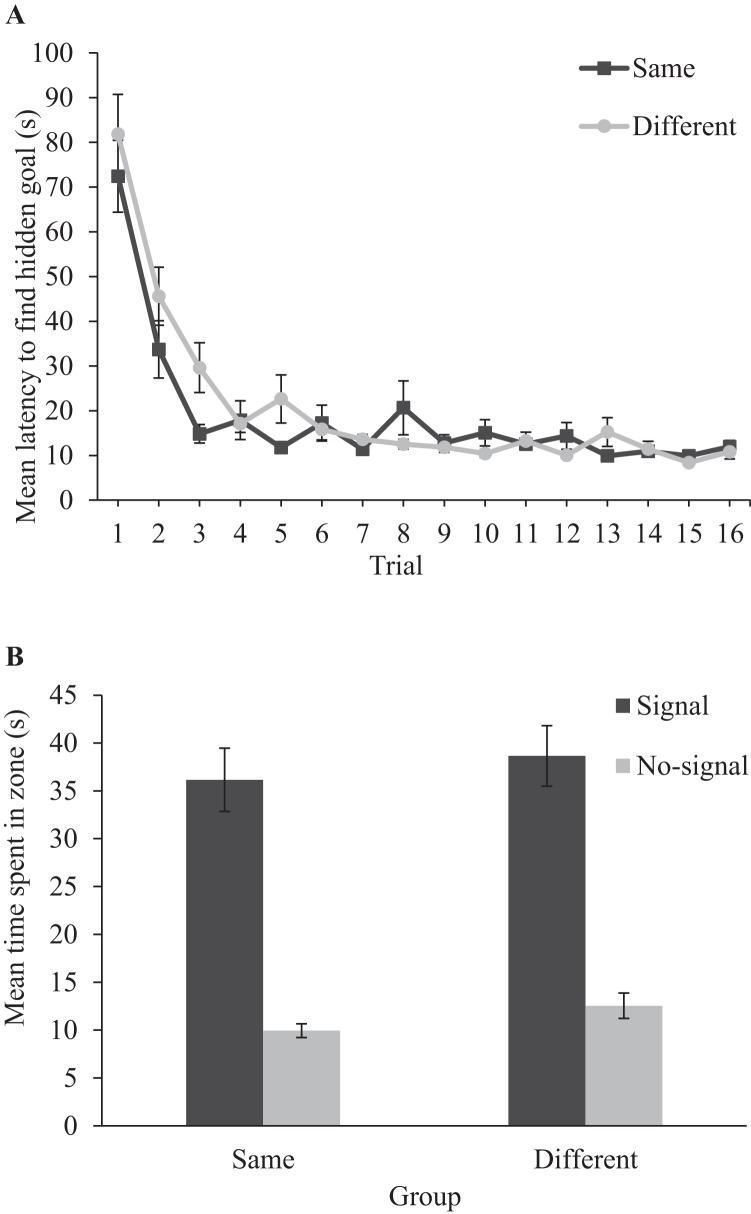
Experiment 1 data. Panel A displays the mean latencies to find the hidden goal during acquisition trials. Panel B displays mean time spent in zones during the test trial for the same and different groups. Error bars show 1 ± standard error of the mean.

Panel A of Figure 2 shows that the latency to find the hidden goal, in seconds, decreased during training in both the same and different groups. A two-way ANOVA conducted on individual latencies to find the hidden goal, with a between-subjects factor of group (same or different), and a within-subjects factor or trial (1–16) revealed only a significant main effect of trial, *F*(15, 450) = 25.91, *MSE* = 354.07, *p* < .001, η_p_^2^ = .46, 90% CI [.39, .49], confirming that participants became quicker to find the hidden goal as training progressed. There was no significant main effect of group, nor a significant interaction between group and trial, both *F*s < 1.[Fig-anchor fig2]

#### Test

Panel B of Figure 2 shows that participants in both the same and different groups preferentially searched in the signal zone over the no-signal zone during test. A two-way ANOVA conducted on individual time spent in zones, with a between-subjects factor of group (same or different), and a within-subjects factor of zone (signal or no-signal) revealed only a significant main effect of zone, *F*(1, 30) = 48.56, *MSE* = 225.49, *p* < .001, η_p_^2^ = .62, 90% CI [.41, .72], confirming that participants spent significantly more time in the signal zone compared with the no-signal zone. There was no significant main effect of group, nor a significant interaction between group and zone, both *F*s < 1.^1^

To assess whether the time spent in zones was different to what would be expected by chance, we expressed the time spent searching in an individual zone as a proportion of the time spent searching in both the signal and no-signal zones, which yielded a chance value of 50%. One-sample *t* tests conducted on individual percentages of time spent in zones revealed that both the same (76%), *t*(15) = 8.69, *p* < .001, *d* = 2.17; and different (73%), *t*(15) = 5.17, *p* < .001, *d* = 1.29, groups spent more time in the correct zone than would be expected by chance. In contrast, the time spent in the no-signal zone was less than chance in both the same (24%), *t*(15) = 8.69, *p* < .001, *d* = 2.17; and different (27%), *t*(15) = 5.17, *p* < .001, *d* = 1.29, groups.

### Discussion

Participants in the current experiment were trained to find a hidden goal on the inside of a kite-shaped arena, before receiving a test trial on the outside of the same arena. For participants in the different group, training and test trials were conducted using environmental textures and colors that rendered the inside and the outside of the arena visually distinct whereas, for participants in the same group, all trials were administered using the same environmental textures and colors. During the test trial, it was observed that participants in both the same and different groups spent more time searching in the signal zone than the no-signal zone, a result that (a) reproduces the effects reported by Buckley et al. (2016b), and (b) suggests both groups relied on global-shape cues to reorient following the perspective change that was caused by the inside-to-outside transfer. Importantly, participants in the same group did not search for any more time at the no-signal zone relative to the different group, indicating that same group participants did not reorient on the basis of local-shape cues to a greater extent than different group participants. Consequently, the current results provide scant support for reorientation based on local-shape cues following a perspective change, even under conditions in which the decrement in generalization suffered by representations of local-shape cues is minimal.

In the current experiment, local-shape cues were defined by the relative wall lengths located in the goal corner (e.g., the left wall being shorter than the right wall). This definition may be overly simplistic, however, because organisms have been observed to reorient on the basis of the angular information provided by the join of two walls (Lubyk et al., 2012; Sturz et al., 2012; Tommasi & Polli, 2004). If local-shape representations comprise both relative wall length and angular information, reorientation based upon local-shape cues during the test phase of the current experiment (and those reported by Buckley et al., 2016b), may not have been apparent because the same local-shape cue that was rewarded during training was simply not present at test. That is, following a transfer from the inside to the outside of a kite-shaped boundary, only the relative wall length information contained within a local-shape representation is preserved, and not the angular information. In order to truly test the notion that reorientation behavior following a perspective change is based only on a global-shape representation, it is necessary to design an environment that preserves both the angular and the relative wall length information of a local-shape representation and, therefore, dissociates behavior based on this local-shape representation from behavior based on a global-shape representation. Experiment 2 was conducted with a novel cross-shaped arena to achieve this.

## Experiment 2

In the Experiments reported by Buckley et al. (2016b), and the current Experiment 1, local-shape information was defined only as the relative wall length information (e.g., McGregor et al., 2006; Pearce, 2009; Pearce et al., 2004). However, both nonhuman (Tommasi & Polli, 2004) and human (Lubyk et al., 2012; Sturz et al., 2012) animals have been noted to reorient using angular information. Consequently, if local-shape information comprises both the relative wall length and angular information provided by the conjunction of two walls, then it is of little surprise that participants do not reorient on the basis of local-shape cues following transfer from the inside to the outside of a kite-shaped arena as the 90° angle of the rewarded corner during training is not preserved at test. The purpose of Experiment 2, therefore, was to adopt a more comprehensive definition of local-shape cues, and assess whether local-shape information could guide reorientation following an inside-to-outside transfer under circumstances in which identical local-shape cues (both angular and relative wall lengths) were present on both sides of the arena.

**Figure 3 fig3:**
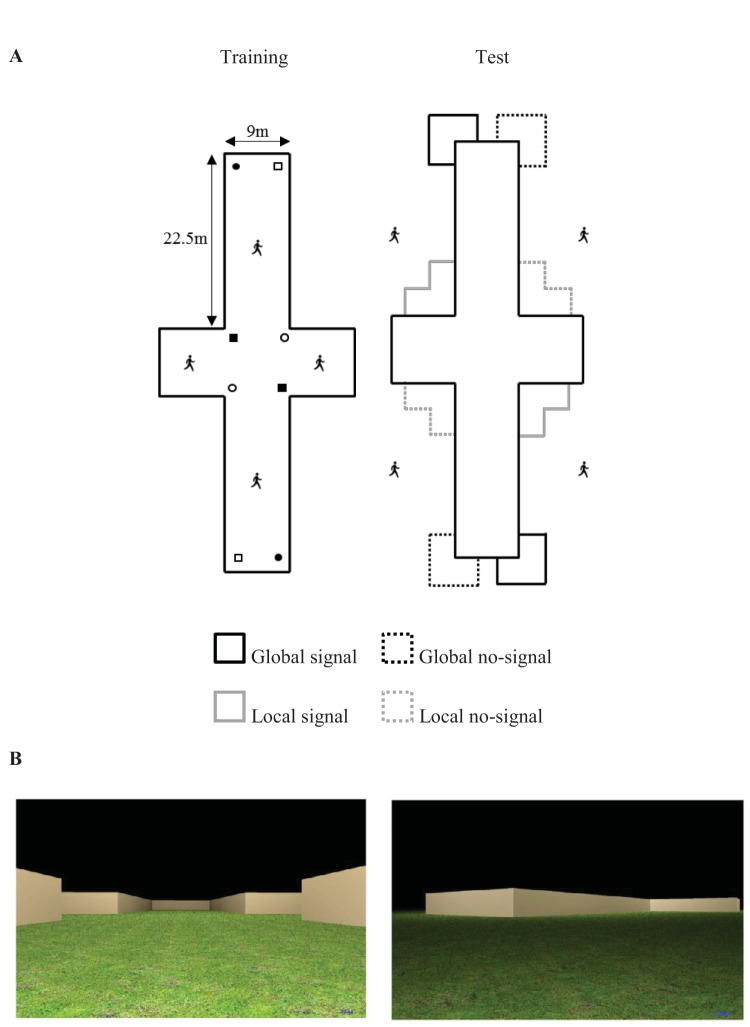
Panel A displays schematic views of the training and test environments used in Experiment 2. Open and closed squares and circles represent the four counterbalanced locations of the hidden goals employed during training. The person inside of the arena indicates the four starting locations used during training for all participants. Square search zones are superimposed on the diagram of the test environment and are labeled with reference to a counterbalancing group for whom the hidden goal was located by the closed circles. The person outside of the arena indicates one of four counterbalanced start locations for the test trial. Panel B displays examples of the training environment inside of the cross-shaped arena (left), and the test environment on the outside of the cross-shaped environment (right).

Participants were trained to find a hidden goal that was located next to a corner within a cross-shaped arena (see Figure 3, Panels A and B), before receiving a test trial, conducted without any hidden goals, on the outside of the same arena. Consider a participant that was trained to find the WiFi signal at an end of one of the long arms of the cross-shaped arena, say, in a right-angled corner where a short wall was to the left of a long wall. When placed on the outside of the environment, the same configuration of a short wall to the left of a long wall, with a 90° corner, is also present. However, this corner is located close to the center of the arena, and is spatially separate from the outside of the corner that contained the goal during training. This arena, then, permits a dissociation of the contribution to reorientation made by local- and by global-shape cues. Crucially, this dissociation can be made when the relative wall lengths *and* angular information that was present on the inside of the arena during training is also present on the outside of the arena at test. Therefore, if reorientation following a perspective transformation can be based upon local-shape information, then participants should search, for at least some time, near the corners on the outside of the arena that were identical to the corner that was rewarded during training on the inside (local signal zone, see Figure 3, Panel A). Alternatively, if reorientation following a perspective transformation is guided only by global-shape representation, we again expect participants to only search at the corners on the outside of the arena that have the closest spatial proximity to those rewarded during training (global signal zone, see Figure 3, Panel A).[Fig-anchor fig3]

### Method

#### Participants

Sixteen students were recruited from the University of Nottingham (nine female), aged between 21 and 33 years (*M* = 24.31, *SD* = 3.44), and were given £5 in return for participation. All participants again provided fully informed consent before commencing the experimental procedure, and the study was ratified by the School of Psychology Research Ethics Committee (University of Nottingham).

#### Materials

Virtual environments that participants viewed from a first-person perspective were again constructed in MazeSuite (Ayaz et al., 2008; www.mazesuite.com), and displayed on the same Apple Macintosh machine used to run Experiment 1. As with the same group of Experiment 1, a grass texture was applied to a 780 m × 780 m floor, and the sky was rendered as a uniform black expanse, when participants were navigating on both the inside and outside of the environment. The cross-shaped environment was built from the same cream-colored walls used in Experiment 1 (see Figure 3, Panel B). Assuming a walking speed similar to that in the real world (2 m/s), the long walls of the cross-shaped environment were 22.5 m long, and the short walls were 9 m long. The height of all walls was approximately 2.5 m. In keeping with Experiment 1, the hidden goals within the arenas were square-shaped regions (1.08 m × 1.08 m) that were always placed 2.48 m away from the walls of the arena, along on a notional line that bisected the corner.

#### Procedure

After signing a standard consent form, participants were given the same standard set of instructions as in Experiment 1. Procedural details for the 16 acquisition trials were identical to Experiment 1, save for the starting location for each trial and the counterbalancing of the goal location. Participants began each trial from one of four positions, located halfway along one of the arms of the cross-shaped environment (see Figure 3, Panel A). The order of start positions was pseudorandomized for each participant, with the stipulation that each of the four start locations was used four times during the 16 acquisition trials, and that consecutive trials never began from the same start location. As with Experiment 1, the direction in which participants began facing was fully randomized for every trial. The hidden goal was located at either a concave or convex corner, with four participants being trained to navigate to find a goal that was located at one of the four positions displayed in Figure 3, Panel A. As with Experiment 1, we wanted to ensure that visits to the correct corner of the cross-shaped environment always resulted in finding the hidden goal. The cross-shaped arena, as with rectangular-shaped arenas, contained two corners that shared the same shape properties; thus, it was necessary for each training environment to contain two hidden goals.

Following training, participants received the same pretest instructions as Experiment 1, and pressing enter began a test trial conducted on the outside of the cross-shaped environment. The cross-shaped environment used in the current experiment was significantly larger than the kite-shaped environment used in Experiment 1; thus, to ensure that participants tested on the outside of the cross-shaped environment had sufficient time to search for the absent hidden goal, we increased the length of the test trials from 120 s to 240 s. Participants began the test trial facing one of the four long walls of the cross-shaped environment, and were located 9 m from the center of that long wall, along a notional line running perpendicular to the wall. There were four possible start locations for the test trial, and each location was used once for every goal location previously described. Navigational behavior at test was measured using the same L-shaped search zones used in Experiment 1. Global-signal zones were located on the outside of the corners where the WiFi signal had been during training, and global-no-signal zones were located at corners that were the mirror image of the global-signal zone corner. Similarly, local-signal zones were located at corners that shared the same local shape information that was rewarded during training, and local-no-signal zones were located at corners that were the mirror of the local-signal zone corner (see Figure 3, Panel A).

### Results

#### Training

**Figure 4:**
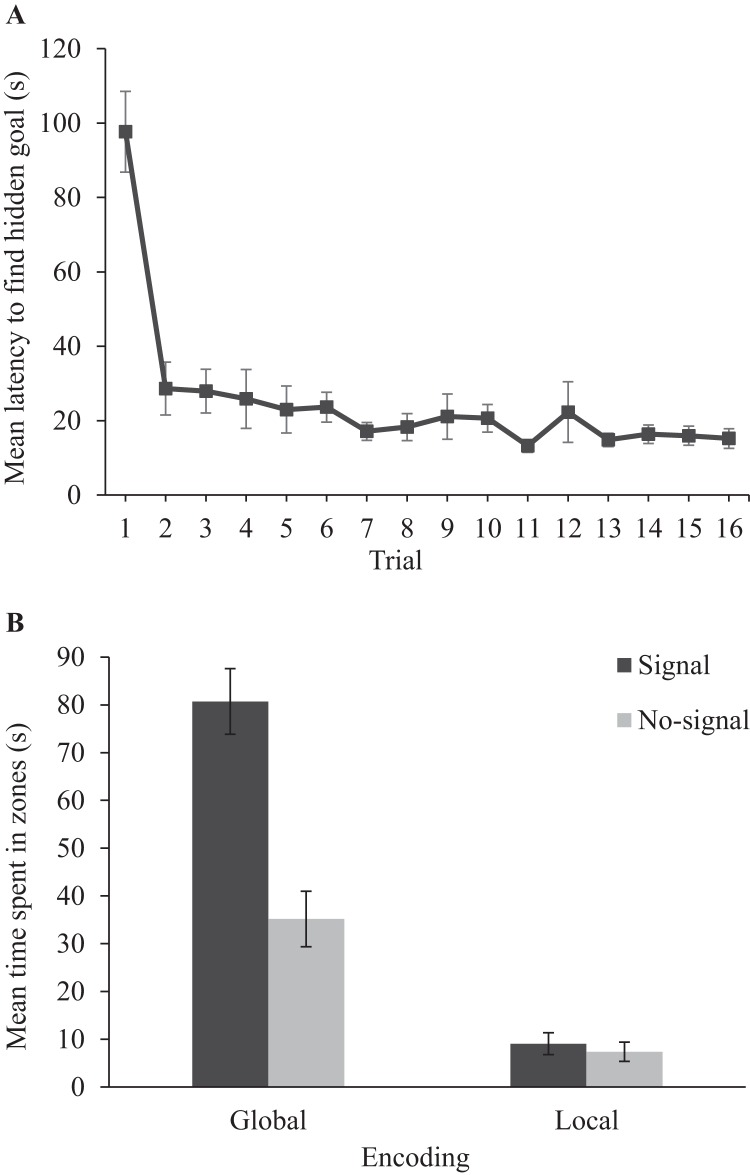
Experiment 2 data. Panel A displays mean latencies to find the hidden goal during acquisition trials. Panel B displays mean time spent in zones during the test trial. Error bars show 1 ± standard error of the mean.

Panel A of Figure 4 shows that the latency to find the hidden goal, in seconds, decreased during training for participants in Experiment 2. A one-way ANOVA conducted on individual latencies to find the hidden goal, with a within-subjects factor of trial (1–16), revealed a significant main effect, *F*(15, 225) = 13.74, *MSE* = 462.30, *p* < .001, η_p_^2^ = .48, 90% CI [.37, .51], confirming that participants became quicker to find the hidden goal as training progressed.^2^

#### Test

Panel B of Figure 4 displays the amount of time that participants in Experiment 2 spent searching within the four measured zones at test. First, participants searched in global zones, per se, to a greater extent than they did in local zones. Second, and more importantly, participants preferentially searched in the global-signal zone over the global-no-signal zone but, in contrast, participants did not preferentially search in the local-signal zone compared to the local-no-signal zone. A two-way ANOVA conducted on individual time spent in zones, with within-subjects factors of encoding (global or local) and zone (signal or no-signal) confirmed these impressions. There were significant main effects of encoding, *F*(1, 15) = 149.27, *MSE* = 265.08, *p* < .001, η_p_^2^ = .91, 90% CI [.80, .94]; and zone, *F*(1, 15) = 16.64, *MSE* = 536.62, *p* = .001, η_p_^2^ = .53, 90% CI [.19, .68], as well as a significant interaction between encoding and zone, *F*(1, 15) = 14.62, *MSE* = 526.00, *p* = .002, η_p_^2^ = .49, 90% CI [.16, .66]. Simple main effects analysis revealed that participants spent more time in the global-signal zone than the local-signal zone, *F*(1, 15) = 74.61, *p* < .001, η_p_^2^ = .83, [.64, .89], and, likewise, more time in the global-no-signal zone compared to the local-no-signal zone, *F*(1, 15) = 25.71, *p* < .001, η_p_^2^ = .63, 90% CI [.31, .76]. More importantly, participants spent more time searching within the global-signal zone compared with the global-no-signal zone, *F*(1, 15) = 15.84, *p* = .001, η_p_^2^ = .51, 90% CI [.18, .68], but spent comparable amounts of time searching in the local-signal and local-no-signal zones, *F*(1, 15) = 1.55, *p* = .23, η_p_^2^ = .09, 90% CI [.00, .33] (see Footnote 3).

To assess whether the time spent in zones was different to what would be expected by chance, we expressed the time spent searching in an individual zone as a proportion of the time spent searching in all four zones (see Figure 3, Panel A), which yielded a chance value of 25%. One-sample *t* tests conducted on individual percentages of time spent in zones revealed that participants spent more time searching in the global signal zone (62%), *t*(15) = 7.09, *p* < .001, *d* = 1.77, than would be expected by chance. In contrast, participants spent no more time searching in global no-signal (26%) zone than would be expected by chance, *t*(15) = .27, *p* = .79, *d* = .07. Finally, participants spent less time searching in both the local signal zone (7%), *t*(15) = 10.32, *p* < .001, *d* = 2.58, and the local no-signal zone (5%), *t*(15) = 13.35, *p* < .001, *d* = 3.34, than would be expected by chance.

### Discussion

Participants in Experiment 2 were trained to find a hidden goal on the inside of a cross-shaped environment before receiving a test trial on the outside of the same environment. Here, participants preferentially searched in the global zones over the local zones. More importantly, it was also the case that participants preferentially searched in the global-signal zone over the global-no-signal zone, but did not display a significant preference for the local-signal zone over the local-no-signal zone. In order to give the best opportunity to detect reorientation based upon local-shape information following an inside-to-outside transformation, Experiment 2 was conducted with an environment that contained the same local-shape information, defined by both relative wall lengths and angular information, on the inside and outside of the environment. In addition, both training and test trials were administered with matching textual features (i.e., arena walls, floors, and sky textures). Even under these conditions, participants were observed to rely only on global-shape information to reorient following a perspective change that was caused by an inside-to-outside transfer. The results of the current experiment, then, provide further evidence that local-shape information is not used for reorientation following a perspective transformation.

## General Discussion

In Experiment 1, participants were trained to find a hidden goal inside a kite-shaped virtual environment, before receiving a test trial conducted on the outside of the same environment. For the different group, training and test trials replicated the procedure and arenas reported in Buckley et al. (2016b). For the same group, the visual information provided by the textures and colors of the arena walls, floors, and skies were equated between training and test. During the test trial, participants in both groups displayed a significant preference for searching in the signal zone over the no-signal zone, indicating that they were reorienting on the basis of global-shape cues, and not local-shape cues. In Experiment 2, participants were again trained to find a WiFi signal, this time on the inside of a cross-shaped environment, before receiving a test trial on the outside of the same environment. During this test, participants displayed a preference for searching in the global signal zone over the global no-signal zone. In contrast, participants scarcely searched in either of the local zones of the environment and also displayed no preference for the local signal zone over the local no-signal zone. Thus, despite administering test trials that contained identical local-shape information on the outside of the arena, participants did not use local-shape information to reorient following the inside-to-outside transfer.

**Figure 5 fig5:**
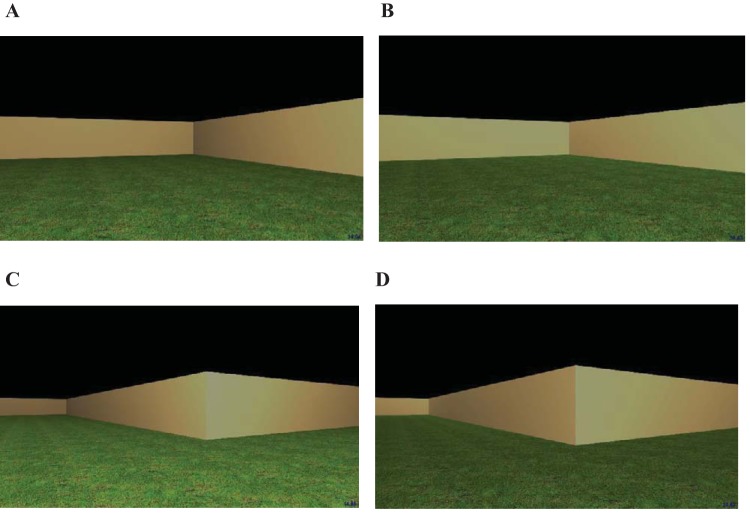
Example views of the cross-shaped maze used in Experiment 2. Panel A: Concave corner viewed from the inside of the maze. Panel B: Concave corner viewed from the outside of the maze. Panel C: Convex corner viewed from the inside of the maze. Panel D: Convex corner viewed from the outside of the maze.

The results of the current experiments, together with those reported by Buckley et al. (2016b), constitute a challenge to accounts of spatial navigation and reorientation that emphasize the role of local-shape encoding (e.g., McGregor et al., 2006; Pearce, 2009; Pearce et al., 2004). Furthermore, these results are also difficult to account for with view-matching analyses of spatial learning (Cheng et al., 2013; Collett & Cartwright, 1983). The results of Experiment 2 are particularly pertinent here, as the exact local-shape information that was present on the inside of the cross-shaped arena was also present on its outside. Given that both angular and relative wall length information contained within a local-shape representation are preserved in this transformation, theories of reorientation based upon view-matching would expect participants to spend at least some time searching in the local signal zone. For instance, consider view-matching theories in which organisms store an image of the environment at a goal location, and navigate by reducing the discrepancy between the currently perceived view and that stored image (e.g., Cheung, Stürzl, Zeil, & Cheng, 2008; Stürzl et al., 2008). Given that the local-signal zone was adjacent to an exterior corner that matched exactly the stored image of the interior corner at the goal location (see Figure 5), view-matching theories would predict that participants would spend more time searching in the local signal zone over the local no-signal zone during the test trial, a result that was not observed. Instead, participants were reorienting based upon a representation of the global-shape of the environment following the perspective change at test.[Fig-anchor fig5]

One objection that may be raised against a global representation analysis of the reorientation behavior we observe is based on the possibility that participants are performing a “mental transformation” at test (see: Meilinger & Vosgerau, 2010; Riecke & McNamara, 2017). For instance, consider a participant trained to find a hidden goal on the inside of the kite-shaped arena in Experiment 1, before receiving a test trial on its outside. Successful reorientation behavior on the outside of the kite could, in principle, be based upon a locally-encoded representation of the goal location, provided that participants can mentally transform the local representation of the corner at the global-signal zone on the outside of the arena, so that it matches the appearance of the same corner from the perspective of the inside of the kite. Although it is difficult to rule out this possibility fully, we have reason to doubt it. In Experiment 2 reported by Buckley et al. (2016b), participants were trained to find a goal on the inside of a kite-shaped environment before, at test, being placing on the outside of a *rectangle-shaped arena*. If reorientation behavior was based on a mental-transformation of the local-shape that was encoded on the inside of the kite-shaped environment, then participants should have searched more at the signal zone than the no-signal zone on the outside of a rectangle-shaped arena, in the same way that they did in Experiment 1 reported here. However, this result was not obtained: Participants displayed no preference for the signal over the no-signal zones during the test trial. This result is, however, consistent with the idea that a global representation of the shape of the environment was encoded during training, and that reorientation based upon this representation was disrupted when the overall shape of the arena was changed at test. Moreover, in order to explain the results of the current Experiment 2, this “mental transformation” analysis would also have to assume that a local representation of the goal location elicited via a transformation (i.e., that elicited by the global-signal zone) was better able to control search behavior than a representation that matched the goal location without any mental transformation (i.e., the local-signal zone). It remains to be determined if this assumption is realistic.

If we accept that the current experiments provide evidence for global encoding of the shape of the environment, then it becomes natural to ask which feature of the global shape is determining search behavior. That is, do participants encode an allocentric global representation of the entire boundary shape, or encode parameters of a global shape such as the principal axis. More colloquially known as the long axis, the principal axis passes, in a kite, from the acute- to the obtuse-angled corners. Cheng and Gallistel (2005) have suggested that organisms can extract the principal axis of an environment and use it to align their global representations of the shapes in which they are navigating. For example, participants in Experiment 1 who received training in which the hidden goal was located in a corner in which the short wall was to the right of a long wall could locate the hidden goal in the kite during training by traveling along the principal axis of the arena, and then heading to the left (see Bodily, Eastman, & Sturz, 2011, for evidence of the role of the principal axis in spatial navigation in adult humans). As we have noted elsewhere (Buckley et al., 2016b), how participants would then use this strategy when transferred to the outside of an environment is rather more complex. For example, if participants had learned to find the hidden goal during training by walking along the principal axis and then turning left, then this strategy would only send participants to the vicinity of the signal zone, at test, if they (a) extrapolated the principal axis beyond the boundaries of the arena (which seems reasonable); and (b) were facing away from the arena as they walked along it. This second assumption, however, may not be reasonable, especially given that participants began each test trial facing *toward* the environmental boundary.

That being said, although our previous embodied description of how participants may use the principal axis to navigate is in-keeping with Cheng and Gallistel’s (2005) descriptions, it is possible to suggest that a more abstract representation of the principal axis could be driving reorientation behavior following an inside-outside transfer, rather than the embodied version we have described above. For example, the goal location may be defined in terms of the principal axis in an allocentric frame of reference. While this analysis may be applied to the experiments presented here, the principal-axis analysis encounters substantial problems with the observation by Buckley et al. (2016b), described previously. Here, training on the inside of a kite was not found to support successful reorientation when testing was conducted on the outside of a rectangle, despite the fact that these two arena shapes can be aligned along their principal axes (Cheng & Gallistel, 2005). With this in mind, and the fact the local shape representations cannot account for search behavior in the current experiments (see also Buckley et al., 2016b, 2019), we propose that reorientation behavior in our inside-to-outside paradigm is controlled by an allocentric representation of the global shape of the environment.

Unlike studies of the role of shape-based navigation and reorientation conducted in nonhuman animals (e.g., Cheng, 1986; Pearce et al., 2004) our studies were conducted using virtual arenas with instructions provided to participants at set points during the procedure. The presence of instructions raises two questions: First, whether their use limits the extent to which the current results can be generalized to nonhuman animals (the so-called “description-experience” gap, Hertwig & Erev, 2009; Madan, Ludvig, & Spetch, 2017). Second, it raises the question of whether they biased participants to behave in a specific manner—such as in a manner that favored the encoding of a global representation of the shape of the environment. For example, participants were instructed prior to the training that the location of the WiFi “*does not move*,” and that prior to the test that its location “*hasn’t changed*” but that the participant would be “*navigating around the outside of the building*.” It is possible that terminology such as this encouraged participants to employ a more global representation of the shape of the environment. However we note that we have used the first two phrases as part of the instructions in experiments that have provided evidence for a *local-encoding* of the shape of the environment (Buckley et al., 2016a, 2016b); thus, instructing participants about the immobility of the hidden goal does not necessarily favor a global navigational strategy.

Previous work in our laboratory has reproduced findings reported by Lew et al. (2014), demonstrating that humans reorient based on local-shape information following shape-transformations between the inside of a rectangle and the inside of a kite (Buckley, Smith, & Haselgrove, 2016a), and we have also observed the same reorientation behavior when both training and test trials are conducted on the outside of these shapes (Buckley et al., 2016b, Experiment 3). There is, however, little evidence that humans rely on a local-shape representation to guide reorientation behavior following a perspective change caused by a transfer from the inside of a boundary to the outside (or vice versa). In both the experiments reported here, and in Experiment 1 reported by Buckley et al. (2016b), participants searched at the corner with the closest spatial proximity to the goal location when transferred across the same-shaped boundary. If the boundary-shape is changed between the training and test phases of an inside-to-outside transfer (e.g., from a kite to rectangle), then participants are unable to reorient, and do not preferentially search at any of the exterior corners at test (Buckley et al., 2016b, Experiment 2). Together, these results suggest that we solely reorient using global-shape representations following a perspective transformation, as reorientation in these circumstances is only successful when training and testing occur with the same-shaped arena. However, it must be noted that, until now, the evidence that humans do not use local-shape information to reorient following a perspective transformation is based largely on a Bayesian analysis that supported the null result reported by Buckley et al. (2016b, Experiment 2). Given this, the results of Experiment 2 in the current article are particularly pertinent. Here, participants scarcely searched in any of the local zones at test, even though these zones were located at corners that matched the rewarded angular and wall length properties from training. Instead, participants preferentially searched at the correct global zone of the cross-shaped arena at test. Consequently, this experiment provides the best support yet to the notion that humans reorient only on the basis of a global representation of shape information following the perspective change that occurs as a result of an inside-outside transfer.

The results presented here, along with previously published studies, are beginning to provide an understanding of the reference frames in which shape-based information is encoded. There is now mounting evidence that humans encode both local (e.g., Buckley et al., 2016a; Lew et al., 2014) and global (e.g., Buckley et al., 2016b) shape-based representations; however, these representations seem to be encoded in different reference frames. As discussed above, local-shape representations have not been observed to guide reorientation behavior following an inside-to-outside transfer, in circumstances in which the angular and relative wall length information is preserved between training and test environments (Experiment 2 of the current paper). Nor is it observed when the global-shape of the arena changes between training and test, which yields a situation where only local-shape representations can be used to reorient, because the learned global-shape representation is now incongruent to the arena shape at test (Buckley et al., 2016b, Experiment 2). However, reorientation based on local-shape cues has been observed following a change in shape, *providing* training and test phases are conducted on the same side of an environmental boundary (Buckley et al., 2016a; Lew et al., 2014; see also Experiment 3 reported by Buckley et al., 2016b). It appears, then, that local-shape representations are not relied upon for reorientation following the perspective change that occurs following a transfer from one side of a boundary to another, and furthermore that this follows from local representations of environmental shape being encoded from a first-person, or egocentric, reference frame. However, as we have demonstrated in the present experiments (see also Experiment 1 of Buckley et al., 2016b), human participants are able to successfully reorient following an inside-to-outside transfer, a behavior we argue to be reliant on a global-shape representation. As this reorientation behavior survived a change in perspective between training and test, it seems reasonable to suggest that this representation is based upon a frame of reference that defines the positions of goal locations with respect to other locations in the environment; that is to say, it is allocentric. The position being advocated here, is that the results of the current experiments, together with those reported by Buckley et al. (2016b) suggest that reorientation can be based on an allocentric, global spatial representation of the environment. We are not ignorant of the fact that this position closely resembles both classical (O’Keefe & Nadel, 1978; Tolman, 1948) and more contemporary (e.g., Doeller & Burgess, 2008) formulations of cognitive mapping theories; however, as we discuss below, the position we advocate is different from cognitive mapping theories in terms of the learning mechanisms that govern allocentric encoding of boundaries.

An enduring debate within the spatial literature concerns the mechanisms by which shape-based information is encoded, and in particular the proposed modularity of encoding a global representation of the shape of the environment (Cheng, 1986; Gallistel, 1990). While others have maintained that shape-based cues can compete with nonshape cues during learning (e.g., Miller & Shettleworth, 2007, 2013; Pearce, 2009), it has been argued by some that learning about environmental boundaries can occur in a manner that is immune to the interference of nonshape cues, such as landmarks (Doeller & Burgess, 2008; Doeller, King, & Burgess, 2008). It is important to note here, that the current experiments speak only to the nature of the shape-based representation, and not to the mechanism by which it is encoded. That is, while the reorientation behavior observed across the two reported experiments is consistent with encoding of a global-shape representation, the data do not allow for comment on whether this representation is encoded in a manner that is immune to interference from nonshape cues. That said, recent experiments conducted in our laboratory have demonstrated that encoding of both local- (Buckley et al., 2016a) and global-shape (Buckley et al., 2019) representations are subject to cue competition from nonshape cues. Traditionally, cue-competition phenomena have been well-explained by domain-general associative learning models (e.g., Miller & Shettleworth, 2007, 2013; Rescorla & Wagner, 1972), but recent theories of navigation based on Bayesian weighting of information (e.g., Xu, Regier, & Newcombe, 2017; see also Cheng, Shettleworth, Huttenlocher, & Rieser, 2007; Ratliff & Newcombe, 2008; Nardini, Jones, Bedford, & Braddick, 2008) will also offer an explanation for the cue competition effects discussed here. To the best of our knowledge, no empirical studies have been designed to test between these two classes of theory, and so future research should address which theoretical framework might offer the best explanation of spatial behavior.

To conclude, in the two experiments reported in this article, participants were trained to find a hidden goal on the inside of an arena, before receiving a test trial on the outside of the same arena. When training and testing were conducted under circumstances in which textural features were matched, or when identical local-shape information was present on both the inside and outside of the environment, participants were observed to reorient on the basis of global-shape information. Together with previous research, these results suggest that humans encode both a representation of local-shape information, and a representation of global-shape information. The challenge, for future research is to determine the theoretical framework that explains how multiple representations of shape and nonshape information combine to control spatial behavior.

## Footnotes

1 During the test trial administered in Experiment 1, some start positions were closer to the signal zone than others. Equally, other start positions were closer to the no-signal zone than others. Given this, is not unreasonable to suggest that some participants may have encountered the signal zone before the no-signal, and other participants the no-signal zone before the signal zone. In the latter case, encountering the no-signal zone before the signal zone may have resulted in local shape representations being better able to compete with global shape representations for control over behavior. To examine this, we reanalyzed the test trial data, including start position (near to local signal zone or far from local signal zone) as an additional factor. This analysis revealed no main effect of start position, and no interactions including start position, *Fs* < 1.

2 It has been demonstrated previously that, at least in chickens, learning and retaining the location of a goal placed at an acute-angled corner is easier compared to learning the location of a goal placed at an obtuse-angled corner (Tommasi & Polli, 2004). To assess this possibility in our experiment, we compared latencies to find the goal during training between participants trained to navigate to an acute- or obtuse-angled corner. A two-way ANOVA conducted on individual latencies to locate the goal, with a between-subjects factor of goal corner (acute or obtuse), and a within-subjects factor of trial (1-16) revealed no main effect of goal corner, *F*(1, 14) = 2.20, *MSE* = 744.96, *p* = .16, or an interaction between goal corner and trial, *F* < 1. In addition, we also incorporated goal corner as an additional factor in our analysis of search data from test trials. A three-way ANOVA conducted on individual time spent in zones, with a between-subjects factor of goal corner (acute or obtuse), and within-subjects factors of encoding (global or local) and zone (signal or no-signal), revealed a significant main effect of goal corner, *F*(1, 14) = 4.78, *MSE* = 95.29, *p* = .047, which indicated that participants trained to find a goal in an acute corner spent significantly less time in the measured zones (*M* = 30.42) compared with participants trained to find a goal in an obtuse corner (*M* = 35.73). Importantly, however, goal corner did not interact with either the encoding or zone factors, and the three-way interaction was also not significant, *Fs*(1, 14) < 1.42, *p*s > .25. Taken together, these analyses reveal that the location of the goal during training had no influence on learning rate, and did not systematically bias search behavior towards any location at test.

3 In the test trial conducted in Experiment 2, some starting positions were between the global and local signal zones, and others were between the global and local no-signal zones. Reference to Figure 3 indicates that, when the goal was located in a concave corner during training, and the test trial began between the two signal zones, the local cue that signaled the goal location was immediately visible to participants. Given this, it might be suggested that, for participants beginning the test trial between the two signal zones, any locally encoded shape information during training might be better able to compete with the globally encoded shape information at test. In addition, when the goal was located at a convex corner during training, participants beginning the test trial between the two signal zones were placed closer to the local signal zone than the local no-signal zone. In keeping with Experiment 1, therefore, is possible that some participants may have encountered the local signal zone before the local no-signal. Encountering the local signal zone before the local no-signal zone may, again, have resulted in local shape representations being better able to compete with global shape representations for control over behavior. To address this, we reanalyzed the test trial data, including start position (near to local signal zone or far from local signal zone) as an additional factor. This analysis revealed no main effect of start position, and no interactions including start position, *F*s < 1.34, *p*s > .27.
